# The dendritic location of the L-type current and its deactivation by the somatic AHP current both contribute to firing bistability in motoneurons

**DOI:** 10.3389/fncom.2014.00004

**Published:** 2014-01-27

**Authors:** Marin Manuel, Daniel Zytnicki, Claude Meunier

**Affiliations:** Laboratoire de Neurophysique et Physiologie, CNRS UMR 8119, Institut des Neurosciences et de la Cognition, Université Paris DescartesParis, France

**Keywords:** bistability, persistent calcium current, afterhyperpolarization, modeling, dynamic clamp

## Abstract

Spinal motoneurons may display a variety of firing patterns including bistability between repetitive firing and quiescence and, more rarely, bistability between two firing states of different frequencies. It was suggested in the past that firing bistability required that the persistent L-type calcium current be segregated in distal dendrites, far away from the spike generating currents. However, this is not supported by more recent data. Using a two compartment model of motoneuron, we show that the different firing patterns may also result from the competition between the more proximal dendritic component of the dendritic L-type conductance and the calcium sensitive potassium conductance responsible for afterhypolarization (AHP). Further emphasizing this point, firing bistability may be also achieved when the L-type current is put in the somatic compartment. However, this requires that the calcium-sensitive potassium conductance be triggered solely by the high threshold calcium currents activated during spikes and not by calcium influx through the L-type current. This prediction was validated by dynamic clamp experiments *in vivo* in lumbar motoneurons of deeply anesthetized cats in which an artificial L-type current was added at the soma. Altogether, our results suggest that the dynamical interaction between the L-type and afterhyperpolarization currents is as fundamental as the segregation of the calcium L-type current in dendrites for controlling the discharge of motoneurons.

## Introduction

The discovery of a L-type calcium current in the dendrites of motoneurons (Schwindt and Crill, 1984; Hounsgaard and Kiehn, 1985) greatly changed our vision of their excitability properties. The steep secondary range of the current-frequency curve was ascribed to its activation (Li et al., 2004), and this persistent calcium current may also trigger dendritic plateau potentials and induce bistability (Hounsgaard and Mintz, 1988; Hounsgaard and Kiehn, 1989), which is defined as the existence of two stable output states for the same input. When bistability occurs between quiescence and firing, motoneurons keep on firing after a depolarizing current pulse instead of going back to rest. Firing bistability, where two stable firing states coexist, was first demonstrated in decerebrate cats in which descending monoaminergic pathways were tonically active (Hounsgaard et al., 1988). When a depolarizing pulse was superimposed on a bias current, the motoneurons fired at higher frequency after the pulse than before.

Plateau potentials and bistability were initially thought to arise from a L-type calcium current located rather distally in dendrites. The presence of a hysteresis in the current-voltage curve in voltage-clamp experiments and the voltage threshold for initiating plateau potentials suggested that the inward current resided in the unclamped portions of the dendritic tree (Lee and Heckman, 1998b). Accordingly, Booth et al. (1997) introduced a model (BRK model hereafter) with two weakly coupled compartments, a spike initiation region and a distal dendritic compartment where the calcium L-type current could initiate a plateau potential, to account for firing bistability. Passive voltage attenuation from soma to dendrites reaches 70% in this model, which would correspond to a distance from the soma of 1.2 times the space constant λ in an equivalent cable. This does not fit with later immunocytochemical and modeling studies (Simon et al., 2003; Elbasiouny et al., 2005, 2006; Ballou et al., 2006; Bui et al., 2006; Zhang et al., 2006; Grande et al., 2007; Zhang et al., 2008), which suggest that the L-type current is closer to the soma (0.6 ± 0.2 λ) and also displays a smaller somatic component. This more proximal location allows the somatic afterhyperpolarization (AHP) following spikes to deactivate the L-type current. Elbasiouny et al. (2006) pointed out that the AHP could enable graded activation of that current in response to synaptic excitation of the dendrites.

This raises the question of the mechanisms underlying motoneuron bistability, and notably firing bistability. Are the same mechanisms at work for the proximal and distal components of the L-type current? Under what conditions is firing bistability achieved? Is some spatial segregation of currents necessary, or can it occur even when the two currents are colocalized? Does the interaction between the L-type and AHP currents play a major role in controlling the firing pattern? We address these issues using a BRK-like model with a stronger coupling between compartments that better matches the location of the bulk of the L-type current in dendrites and allows it to interact with the somatic AHP current. We demonstrate that the dynamical interaction between these currents conditions the firing pattern. To emphasize this point, we show that a somatic L-type current may also lead to firing bistability. However, this requires that the AHP conductance be solely activated by the high-voltage-activated calcium conductances turned on during action potentials. Using dynamic clamp, we mimicked experimentally that condition in motoneurons of anesthetized cats and validated our theoretical prediction. Altogether, our study indicates that, in addition to the spatial segregation of most of the calcium L-type current in the dendrites, the competition between the somatic AHP current and the L-type current largely determines the firing pattern. In particular, firing bistability is achieved when a large AHP current counterbalances a strong L-type calcium current. The spatial segregation acts by increasing the hysteresis of the current-frequency (*F-I*) curve and the firing bistability that are created by the dynamical interaction between the somatic AHP current and the L-type current.

## Materials and methods

### Model

**Figure 1:**
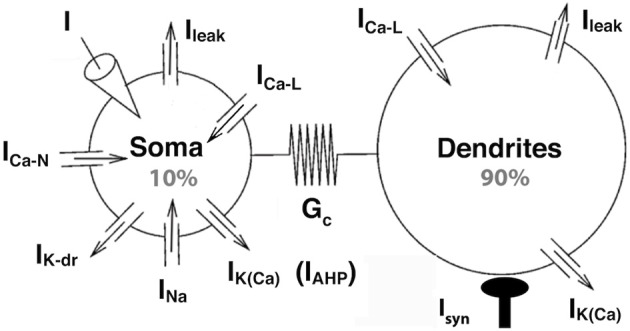
**The strong coupling model**. The two compartments have similar passive properties, and they are coupled via a symmetrical conductance ten times larger than in the BRK model. The soma is endowed with spike generating currents and an AHP current. A L-type calcium current is present in the soma (somatic variant of the model) or in the dendrites (dendritic variant). The dendritic component of the N-type calcium current was eliminated from the BRK model, and the activation curve of the somatic component is steeper (see text). Dendrites are endowed with a calcium sensitive potassium conductance. Input consists in either a current injected in the soma or synaptic excitation of the dendrites.

Our model has the same bi-compartmental structure as the BRK model and the same complement of currents (see Figure 1). Briefly, the membrane areas of the somatic and dendritic compartments represent *p* = 10% and 1 − p = 90% of the total membrane surface *S*, respectively. They are electrically coupled via the passive conductance *G_c_S*. We set the coupling conductance *G_c_* to 1.0 mS/cm^2^, one order of magnitude larger than in the BRK model. We adopted a symmetrical coupling, i.e., the same coupling conductance *G_c_* from soma to dendrites and from dendrites to soma, and assumed that soma and dendrites had the same specific leak conductance *G*_leak_, as in the BRK model.

The somatic compartment of our model is endowed with transient sodium and delayed rectifier potassium currents, responsible for the generation of action potentials. A fast activating and slowly inactivating N-type current allows calcium to enter the soma during action potentials as in the BRK model. A non-inactivating L-type calcium current *I*_*Ca* − *L*_ = *G*_*Ca* − *L*_ *m*_*Ca* − *L*_ (*V_Ca_* − *V_S_*) is added to the soma in the second part of our study. Here, *V_S_* is the somatic current, *V_Ca_* the reversal potential of the calcium currents, *G*_*Ca*−*L*_ the maximal conductance of the L-type current and *m*_*Ca* − Ł_ its activation variable of the current. Elevation of the calcium concentration in the soma triggers the calcium-sensitive potassium current *I*_AHP_ = *G*_AHP_ [*Ca*^2+^]/([*Ca*^2+^]+*K_Ca_*)(*V_K_* − *V_S_*), of maximum conductance *G*_AHP_, reversal potential *V_K_* and half-activation calcium concentration *K_Ca_*. This current is responsible for the AHP following spikes (hence the “AHP” subscript that allows to distinguish this current from the dendritic calcium-activated potassium current). We steepened the activation curve of the somatic N-type calcium current compared to the BRK model (1 mV width vs. 5 mV) so that this AHP conductance is not activated before firing onset.

In the first part of our study, dendrites, whose voltage is denoted by *V_D_*, are endowed with a L-type calcium current, the activation of which can trigger plateau potentials, and a calcium sensitive potassium current *I*_*K*(*Ca*)_ = *G*_*K*(*Ca*)_[*Ca*^2+^]/([*Ca*^2+^]+*K_Ca_*)(*V_K_* − *V_D_*). We removed the dendritic N-type calcium current of the BRK model as it was not activated enough to affect significantly the behavior of the model. Finally, we hyperpolarized the reversal potential of the leak current from −60 to −70 mV in both soma and dendrites. The value of −60 mV was required in the original BRK model to allow the current injected in the soma to depolarize substantially the dendrites in spite of the weak coupling. In contrast, they are too easily depolarized in our strongly coupled model if *V*_leak_ is set at −60 mV. That is why we adopted a more hyperpolarized value. All differences with the BRK model are summarized in Table 1. All other parameters are the same as in Booth et al. (1997).

**Table 1:** **Differences between the BRK model and our model**.

	**BRK model**	**Our model**
Coupling conductance	0.1 mS/cm^2^	1.0 mS/cm^2^
Leak reversal potential	−60 mV	−70 mV
Dendritic N-type conductance	0.3 mS/cm^2^	None
Activation of somatic N-type current	1/(1 + exp(−(*V* + 30)/5)), V in mV	1/(1 + exp(−(*V* + 30)))
Dendritic potassium conductance	Always 0.7 mS/cm^2^	Set to 0 or 0.7 mS/cm^2^

We set the dendritic potassium conductance G_K(Ca)_ to 0 in most of our study and accordingly decreased G_Ca-L_.

Except for these differences, the gating variables of the voltage-dependent currents follow the same first order kinetic equations as in Booth et al. (1997). The evolution of the calcium concentration in a compartment is modeled by the phenomenological equation τ*_Ca_d*[*Ca*^2+^]/*dt* = α_*N*_ *I*_*Ca* − *N*_ + α_*L*_ *I*_*Ca* − *L*_ − [*Ca*^2+^] where calcium ions influx through the L- and N-type conductances, quantified by α_*L*_ and α_*N*_ respectively, is counterbalanced by buffering (Booth et al., 1997). The differential equations describing the model are solved using a standard fourth order Runge-Kutta algorithm.

Our model does not incoporate all the ionic currents known to exist in motoneurons. As in the BRK model, no hyperpolarization-activated *I_h_* current was added, because this current would contribute little to the repetitive discharge. Similarly, we did not introduce a persistent sodium current (Kuo et al., 2006) since robust repetitive firing was elicited in the model without it. Finally, we decided to not incorporate voltage-dependent potassium conductances in dendrites after checking that they counterbalanced the calcium L-type in the same way as calcium-sensitive potassium channels. These deviations from realism are of limited significance as our emphasis is on the interplay between calcium and calcium-sensitive potassium currents. We also note that the Cav1 channels underlying the L-type current may display facilitation properties, as demonstrated by Moritz et al. (2007) in rat hypoglossal motoneurons, which might have some impact on hysteretical properties of motoneurons. They are not incorporated in the present model.

We investigated the response of the model (current-frequency curve, conductance-frequency curve, activation of the L-type current) to triangular ramps of current injected in the soma and ramps of tonic synaptic excitation of the dendrites. Tonic synaptic excitation of dendrites was modeled by a current *I_D_* (*t*) = *G*_syn_ (*t*)(*V*_syn_ − *V_D_*(*t*)) with slowly varying conductance *G*_syn_(*t*) and reversal potential *V*_syn_ = 0 mV. The model behavior was strongly affected by the ramp velocity. We had to use very slow ramps (velocity of 0.01–0.1 nA/cm^2^.s) to be sure that the hysteresis of the F-I curve did not arise from memory effects.

Finally, we note that Kim et al. (2009) showed, using two-ports circuit theory (Jaffe and Carnevale, 1999), that a two compartments model could account for the bi-directional voltage attenuation between the soma and some given location in dendrites, if the coupling between compartments was assymetrical or if the two compartments had different specific conductance. However, these two compartments do not map to the soma and the denditic tree. Therefore, such models are unsuitable for our purpose that is to study how active currents located in the soma and in the dendrites interact.

### Dynamic clamp experiments

Experiments were carried out on four adult cats (3.9–4.4 kg) deeply anesthetized with sodium pentobarbitone (Pentobarbital, Sanofi). In accordance with French legislation, the investigators had a valid license to perform experiments on live vertebrates delivered by the Direction des Services Vétérinaires (Préfecture de Police, Paris). The animal house and the experimental room had received the agreement of the same authority. Anesthesia was induced with an intraperitoneal injection (45 mg·kg^−1^), supplemented whenever necessary (usually every 2 h) by intravenous injections (3–6 mg·kg^−1^). Animals were paralyzed with Pancuronium Bromide (Pavulon, Organon SA) at a rate of 0.4 mg·h^−1^ and artificially ventilated (end tidal pCO_2_ maintained around 4%). A bilateral pneumothorax prevented movements of the rib cage. The adequacy of anesthesia was assessed on myotic pupils and on the stability of blood pressure (measured in the carotid) and of heart rate. At the onset of experiment, amoxicillin (500 mg; Clamoxyl, Merieux) and methylprenidsolone (5 mg; Solu-Medrol, Pharmacia) were given subcutaneously to prevent the risk of infection and edema, respectively. The central temperature was kept at 38°C. Blood pressure was maintained above 90 mmHg by perfusion of a 4% glucose solution containing NaHCO_3_ (1%) and gelatin (14%; Plasmagel, Roger Bellon) at a rate of 3–12 ml·h^−1^. A catheter allowed evacuation of urine from the bladder. At the end of the experiments, animals were killed with a lethal intravenous injection of pentobarbitone (250 mg).

The following nerves were cut, dissected and mounted on a pair of stimulating electrodes to identify recorded motoneurons: anterior biceps and semi-membranosus taken together (ABSm), the gastrocnemius medialis together with gastrocnemius lateralis and soleus nerves (Triceps surae, TS), the remaining part of the tibialis nerve (Tib), the common peroneal nerve (CP), and the posterior biceps and semitendinosus taken together (PBSt). The lumbosacral spinal segments were exposed by laminectomy, and the tissues in hind limb and spinal cord were covered with pools of mineral oil kept at 38°C.

Intracellular recordings of motoneurons were made using micropipettes (tip diameter 2.0–2.5 μm) filled with KCl 3 M (resistance 2–4 MΩ) and an Axoclamp 2B amplifier (Molecular Devices, Sunnyvale, USA) connected to a Power1401 interface using the Spike2 software (CED, Cambridge, UK). After impalement, identification of motoneurons rested on the observation of antidromic action potentials in response to the electrical stimulation of their axon in a peripheral nerve. All motoneurons retained for analysis had a resting membrane potential more hyperpolarized than 50 mV, which varied by less than 5 mV over the recording session. The axonal conduction velocity was computed from the latency of the antidromic action potentials.

Dynamic clamp recordings were done using the Discontinuous Current Clamp mode (7–9 kHz) of the amplifier because it allows for reliable measurements of the membrane potential, even when large currents are injected (Brizzi et al., 2004 see also Prinz et al., 2004). A dynamic clamp current *I_DC_* = *G_DC_ m_DC_*(*V_DC_* − *V_S_*) of maximum conductance *G_DC_*, activation variable *m_DC_* and reversal potential *V_DC_*, mimicking a L-type calcium current, was injected into the motoneuron soma through the recording micropipette. The activation variable evolved according to τ_*DC*_ *dm_DC_*/*dt* = *m*_∞_(*V_S_*) − *m_DC_* where the steady-state activation function is *m*_∞_(*V_S_*) = 1/(1+exp((*V* − θ*_DC_*)/*k_DC_*)), τ_*DC*_ is the activation time constant, θ_*DC*_ is the half activation voltage and *k_DC_* determines the slope of the activation curve. This artificial L-type current was computed at a speed of 10 kHz by a PC running the real time RTLinux kernel and the dynamic clamp software MRCI (software MRCI, Raikov et al., 2004).

## Results

### Increasing the coupling suppresses the firing bistability of the BRK model

The (somatic) input conductance *G_in_* = (*G_S_*/*p*)(*G_C_* + *p*(1 − *p*)*G*_leak_)/(*G_C_* + (1 − *p*)*G*_leak_) of our model is 7.8 times the conductance *G_S_* = *G*_leak_*pS* of the soma itself, thrice more than in the BRK model (2.6). This is more in keeping with the ratio of dendritic dominance of motoneurons, i.e., the ratio of the input conductance at the soma to the soma conductance, which is typically of the order of 10 (Fleshman et al., 1988). Passive steady-state voltage attenuation from the soma to the dendrites *G*_leak_ /(*G*_leak_ + *G_C_* /(1 − *p*)) is 35%, which corresponds to a distance of 0.43 λ from the soma on an equivalent cable and fits better with the location of the L-type current in dendrites than in the BRK model. This is because the dendritic compartment represents only the distal part of dendrites in the BRK model, all the proximal part being modeled by the low coupling conductance between the two compartments.

**Figure 2:**
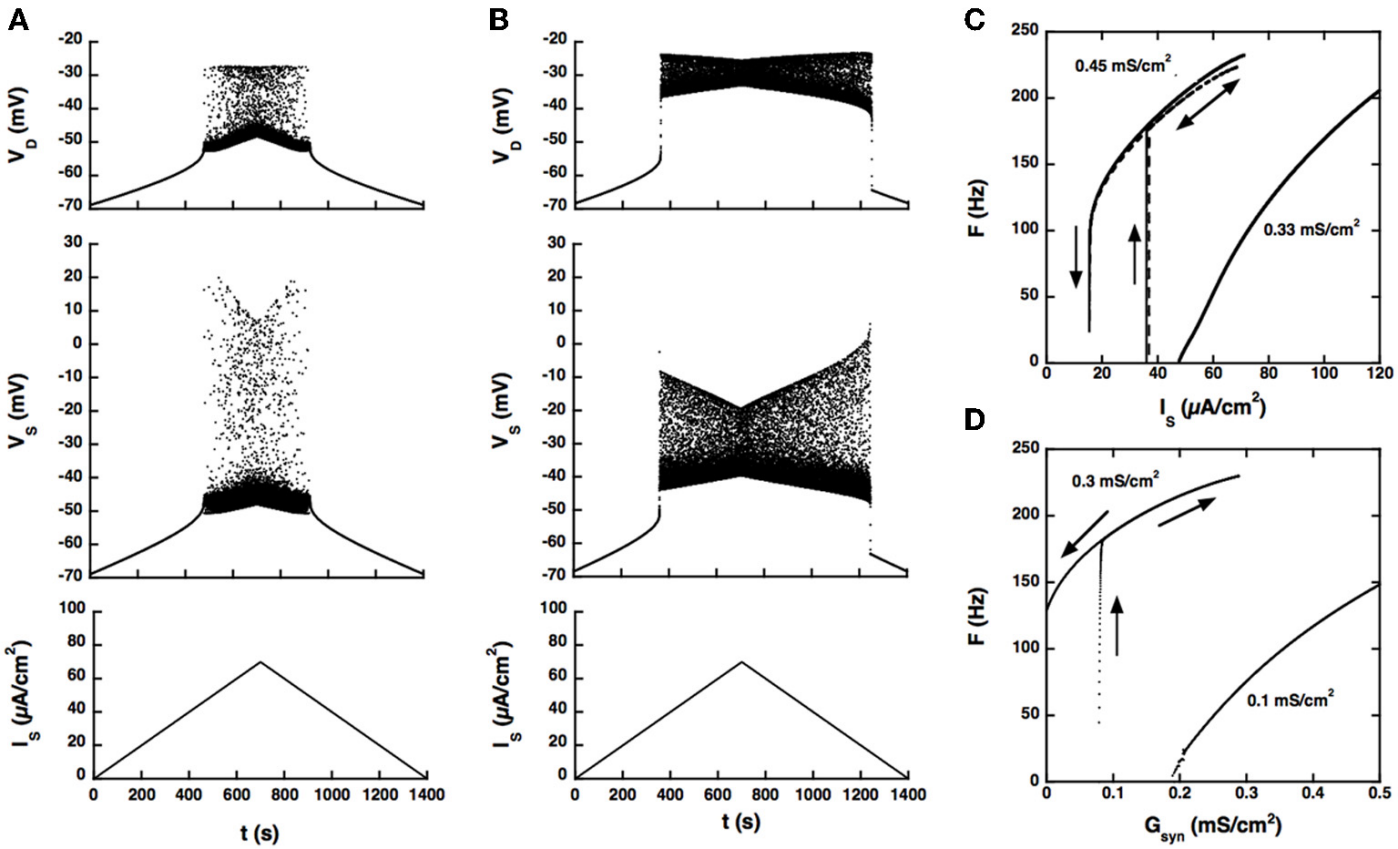
**Response of the strong coupling model (*G_c_* = 1 mS/cm^2^) to excitatory input. (A)** Voltage response to a triangular current ramp. Dendritic *G*_*Ca*−*L*_ is 0.33 mS/cm^2^ as in the BRK model. From bottom to top: current injected in the soma, soma voltage and dendritic voltage. The discharge is symmetrical, the recruitment current on the ascending ramp and the derecruitment current on the descending ramp differing by less than 1.5% (50.2 and 49.4 mV, respectively). **(B)** Same as A but *G*_*Ca*−*L*_ increased to 0.45 mS/cm^2^. The discharge is clearly asymmetrical. A dendritic plateau potential of 16 mV sets in at firing onset (*I_S_* = 38 μA/cm^2^). On the descending ramp, firing persists down to 20 μA/cm^2^. **(C)** *F-I* curves. Current ramp from 0 to 120 μA/cm^2^ and back. *F-I* curves (solid lines) are displayed for *G*_*Ca*−*L*_ = 0.33 (right), and 0.45 mS/cm^2^ (left). For this latter value,firing stops when the injected current reaches 70 μA/cm^2^ because of spike blockade. Decreasing the dendritic *G*_*K*(*Ca*)_ from 0.7 to 0.25 mS/cm^2^ (with *G*_*Ca*−*L*_ kept at 0.33 mS/cm^2^, dashed line) has the same effect as increasing *G*_*Ca*−*L*_ from 0.33 to 0.45 mS/cm^2^ [with *G*_*K*(*Ca*)_ kept at 0.7 mS/cm^2^]. **(D)** Synaptic excitation of dendrites. *F-G*_syn_ curves are shown for *G*_*Ca*−*L*_ = 0.1 (right) and 0.3 mS/cm^2^ (left). No dendritic potassium conductance *G*_*K*(*Ca*)_. Triangular conductance ramp from 0 to 0.5 mS/cm^2^ (i.e., equal to the leak conductance of dendrites) and back, velocity of 0.01 mS/cm^2^/s. Note that frequency plateaus are present near firing onset for *G*_*Ca*−*L*_ = 0.1 mS/cm^2^ as in the subprimary firing range of mouse motoneurons (Manuel et al., 2009). We showed that they are due to mixed mode oscillations in a previous paper (Iglesias et al., 2011). The ascending (upward pointing arrows) and descending branches (downward pointing arrows) of the hysteresis loops are indicated on panels **(C,D)** and on the following figures.

The firing bistability observed in the BRK model for standard parameters (dendritic *G*_*Ga* − *L*_ = 0.33 mS/cm^2^, dendritic *G*_*K*(*Ca*)_ = 0.7 mS/cm^2^, *G*_AHP_ = 3.14 mS/cm^2^) disappears when the coupling conductance *G_c_* is increased to 1 mS/cm^2^. A slow triangular ramp of current injected in the soma (from 0 to 70 μA/cm^2^ and back with a velocity of 10^−4^ μA/cm^2^.s) elicits a symmetrical discharge, as shown in Figure 2A. The *F-I* curve is graded, the firing rate linearly increasing from 5 Hz at recruitment to 100 Hz for an injected current of 75 μA/cm^2^ (see Figure 2C) and then progressively saturates.

When the L-type current is increased to compensate for the larger input conductance, quiescence/firing bistability is achieved, but firing bistability cannot be recovered. This is illustrated in Figure 2B for *G*_*Ca* − *L*_ = 0.45 mS/cm^2^. The discharge becomes asymmetrical and exhibits a large domain of bistability between quiescence and firing, as shown by the *F-I* curve (Figure 2C, right trace). On the ascending ramp, firing starts at high frequency (175 Hz). On the descending ramp, the discharge persists well below the recruitment current.

Only these two firing regimes are observed. No firing bistability occurs for intermediate values of *G*_*Ca* − *L*_. We also note that decreasing the potassium conductance in dendrites has the same effect as increasing the L-type current, as illustrated in Figure 2C (dashed line). *G*_*K*(*Ca*)_ can even be set to 0 provided that the L-type current is appropriately decreased.

Increasing *G*_*Ca* − *L*_ has the same effect on firing when the drive is provided by synaptic excitation of dendrites, as shown in Figure 2D. For *G*_*Ca* − *L*_ = 0.1 mS/cm^2^ and no potassium conductance in dendrites, firing starts at 5 Hz and the conductance-frequency curve is graded. In contrast, for *G*_*Ca* − *L*_ = 0.3 mS/cm^2^, firing starts at 165 Hz and the *F-G*_syn_ curve displays a large range of bistability between quiescence (on the ascending ramp) and firing (on the descending ramp). No firing bistability is observed at any intermediate value of *G*_*Ca* − *L*_.

### Firing bistability requires large dendritic *G*_*Ca* − *L*_ and somatic *G*_*K*(*Ca*)_

**Figure 3:**
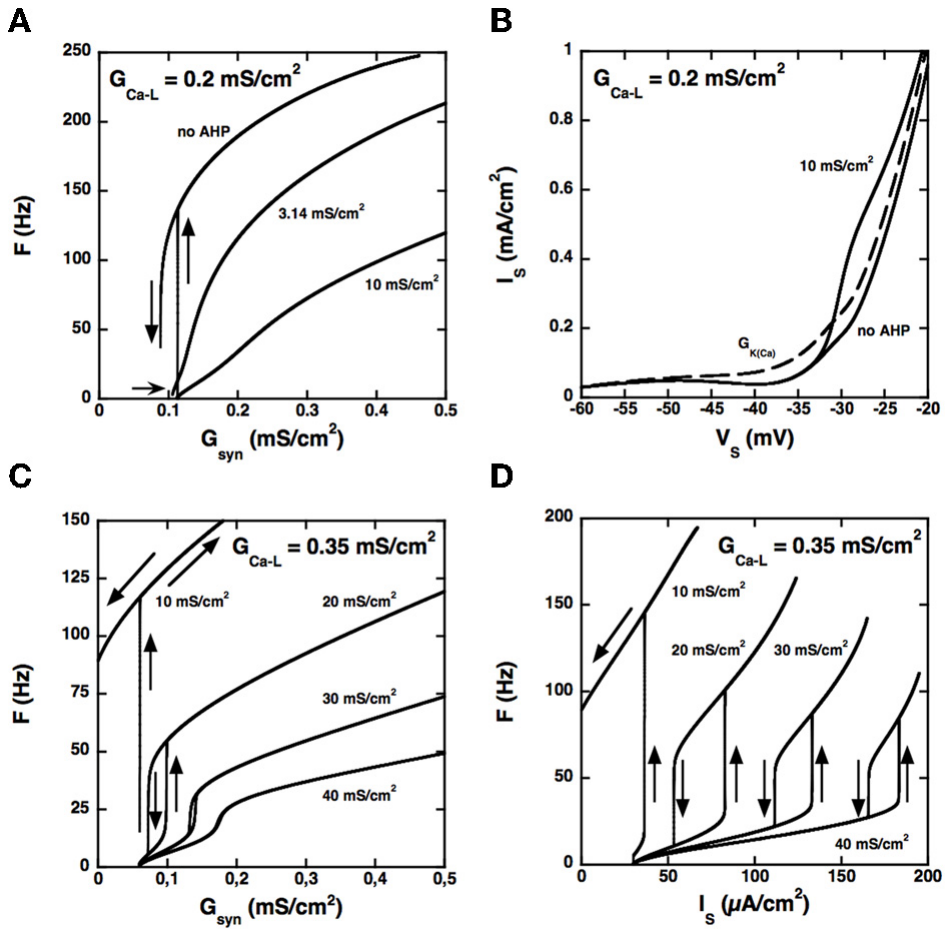
**Control of the firing pattern by the AHP conductance. (A)** *F-G*_syn_ curves for *G*_*Ca* − *L*_ = 0.2 mS/cm^2^. Somatic AHP conductance *G*_AHP_ = 0.0, 3.14, and 10 mS/cm^2^ (see labels) but no potassium conductance in dendrites. For *G*_AHP_ = 3.14 mS/cm^2^ (the same value as in the BRK model), the *F-G*_syn_ curve is graded and displays only a tiny range of bistability below recruitment (thin arrow). When the AHP is suppressed, firing starts at 137 Hz, and the size of the bistability range considerably increases. In contrast, bistability disappears when *G*_AHP_ is increased to 10 mS/cm^2^. (**B)** Somatic I–V curves. Same conditions as in **(A)**. The curves obtained without AHP and for *G*_AHP_ = 10 mS/cm^2^ (solid lines, see labels) differ only above −33 mV. In contrast, increasing the dendritic *G*_*K*(*Ca*)_ to 0.7 mS/cm^2^ (in the absence of somatic AHP) suppresses the negative slope region of the I–V curve in the subthreshold voltage range [dashed line labeled *G*_*K*(*Ca*)_]. The curves are not hysteretic because a dendritic compartment strongly coupled to the soma cannot account for the distal dendritic component of the L-type current. This departure from realism is of little significance as we focus here on the interaction between the proximal component of the L-type current and the somatic AHP. **(C)** *F-G*_syn_ curves for *G*_*Ca* − *L*_ = 0.35 mS/cm^2^. *G*_AHP_ = 10, 20, 30, and 40 mS/cm^2^ (see labels). At variance with panel A, the *F-G*_syn_ curves display a primary range of firing when *G*_AHP_ is large enough to counterbalance the L-type current and has then a strong regulatory effect on the discharge. **(D)** *F-I* curves for *G*_*Ca* − *L*_ = 0.35 mS/cm^2^. Same as in D but current is injected in the soma. Note that the large AHPs at play create a wide primary firing range, in keeping with the traditional role of the AHP in firing rate control.

Because the two compartments are strongly coupled, the AHP triggered by spikes at the soma may produce a sufficient hyperpolarization of dendrites to hinder activation of the L-type current, in line with the results of Elbasiouny et al. (2006). As a consequence, increasing the AHP prevents high frequency firing at discharge onset and suppresses quiescence/firing bistability, as shown in Figure 3A for *G*_*Ca* − *L*_ = 0.2 mS/cm^2^.

Increasing the somatic AHP conductance is not equivalent to increasing the dendritic *G*_*K*(*Ca*)_ (or decreasing the L-type current). Because the AHP conductance is recruited only during spikes, following the activation of the N-type calcium current, the recruitment threshold is insensitive to *G*_AHP_ (see Figure 3A), whereas it considerably increases with *G*_*K*(*Ca*)_. For the same reason, the AHP current has no impact on the current-voltage (I–V) curve in the subthreshold voltage range, as illustrated in Figure 3B. For *G*_*Ca* − *L*_ = 0.2 mS/cm^2^ the somatic I–V curve is slightly N-shaped in the absence of AHP. Adding an AHP conductance of 10 mS/cm^2^ alters the curve only in the suprathreshold voltage range, so that it remains N-shaped. In contrast, adding a dendritic potassium conductance of 0.7 mS/cm^2^ also modifies the I–V curve in the subthreshold range, and it becomes monotonic.

Although the I–V curve displays a negative slope region for *G*_AHP_ = 10 mS/cm^2^, the membrane voltage exhibits no plateau potential (not shown). Moreover, the synaptic conductance-frequency (*F-G*_syn_) curve is graded and exhibits no hysteresis when the synaptic conductance is decreased back (see Figure 3A). This demonstrates that the disappearance of quiescence/firing bistability is not due a change of the I–V curve from N shaped to monotonic. It results from the dynamical interaction between the L-type current and the AHP current during firing.

Importantly, the interaction between the L-type and AHP conductances can elicit firing bistability when *G*_*Ca* − *L*_ is increased beyond 0.27 mS/cm^2^. This is illustrated in Figure 3C for *G*_*Ca* − *L*_ = 0.35 mS/cm^2^. Quiescence/firing bistability is observed for *G*_AHP_ below 12 mS/cm^2^ (upper curve, *G*_AHP_ = 10 mS/cm^2^). For *G*_AHP_ above 30 mS/cm^2^ (bottom curve, *G*_AHP_ = 40 mS/cm^2^) the *F-G*_syn_ curve smoothly increases from a very low value (1.2 Hz). In between, for *G*_AHP_ ranging from 12 to 30 mS/cm^2^, firing bistability is achieved. This is almost one order of magnitude larger than for the transition from quiescence/firing bistability to graded firing for *G*_*Ca* − *L*_ = 0.2 mS/cm^2^ (see Figure 3A).

Similar results are obtained for current injection in the soma, but the domain of firing bistability is wider than for synaptic excitation of dendrites. For instance, firing bistability is achieved for *G*_AHP_ between 5 and 40 mS/cm^2^ when *G*_*Ca* − *L*_ is set at 0.35 mS/cm^2^ (see Figure 3D), nearly twice more than for synaptic input. The counterclockwise hysteresis of the *F-I* reflects a genuine bistability between two different firing states. Indeed, we also checked that excitatory and inhibitory current pulses could switch the model from the low frequency firing state to the high frequency state and back (not illustrated).

Altogether our results demonstrate that bistability is controlled by the competition between the dendritic calcium L-type current and the somatic AHP current when the two compartments of the model are strongly coupled. This competition did not occur in the weakly coupled BRK model where the AHP was too attenuated in dendrites to deactivate the L-type current. This shows that the strong spatial segregation between the L-type current and the currents underlying the discharge (transient sodium, delayed rectifier and AHP currents) present in the BRK model is not necessary for achieving firing bistability.

### A somatic L-type calcium current may elicit firing bistability by itself

Our model suggests that the firing pattern of motoneurons is largely determined by the interaction between the AHP and L-type currents. To further test this hypothesis, we examined whether the competition between the AHP conductance and a somatic L-type current could also lead to bistability. Accordingly, we suppressed the dendritic component of the L-type current and incorporated instead a somatic L-type current in the model. As before, no potassium current was present in the dendrites. Dendrites were thus passive, all active conductances being confined to the axo-somatic compartment.

**Figure 4:**
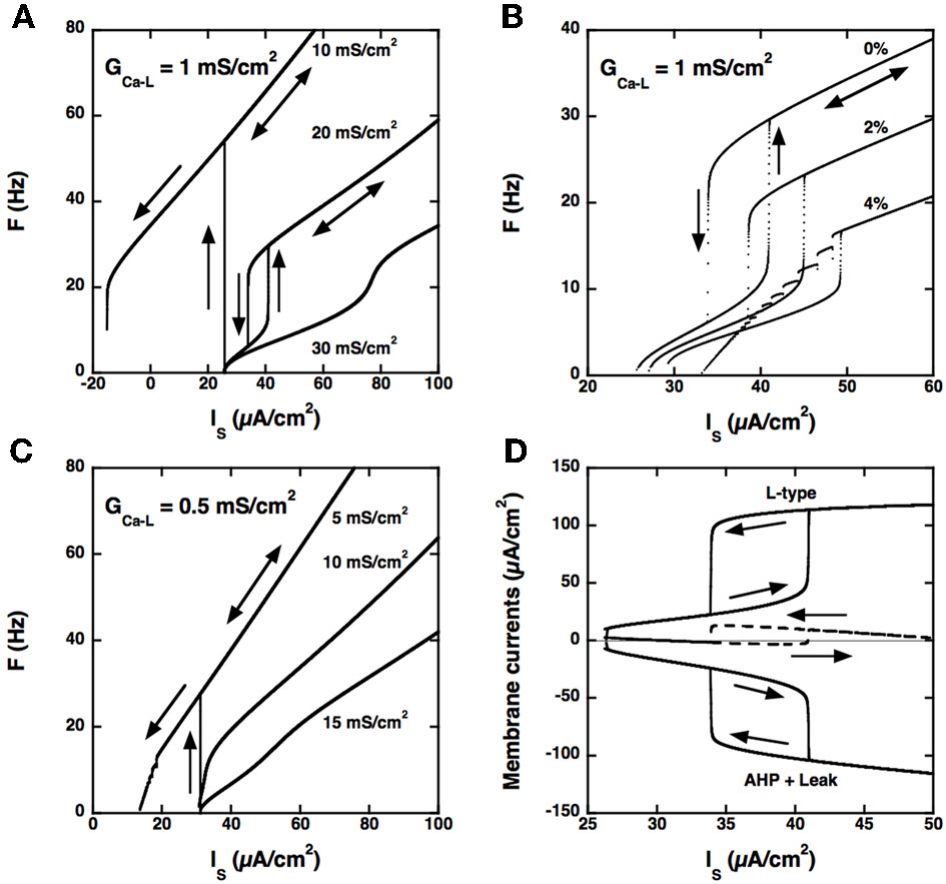
**Model with somatic L-type current and passive dendrites. (A)** *F-I* curves for *G*_*Ca* − *L*_ = 1.0 mS/cm^2^. Triangular ramp of current injected in the soma from 0 to 100 μA/cm^2^ and back (velocity: 10^−4^ μA/cm^2^.s). Increasing values of *G*_AHP_ from top to bottom, see labels). α_*N*_ = 0.0045 mol/nA.cm as before, but α_*L*_ is set to 0. When *G*_AHP_ = 10 mS/cm^2^ (top curve), firing starts at 54 Hz at the recruitment threshold (32 μA/cm^2^), and the *F-I* curve shows a large domain of quiescence/firing bistability, derecruitment occuring only for a negative current of −15 μA/cm^2^ on the descending ramp. Firing bistability is observed for between 15 and 25 (middle curve, *G*_AHP_ = 20 mS/cm^2^). When *G*_AHP_ is increased beyond 25 mS/cm^2^, bistability disappears and the *F-I* curve becomes graded (bottom curve, *G*_AHP_ = 30 mS/cm^2^). Note that the recruitment threshold remains unchanged. **(B)** Effect of α_*L*_ on firing. *F-I* curves for *G*_*Ca* − *L*_ = 1 mS/cm^2^ and *G*_AHP_ = 20 mS/cm^2^ and α_*N*_ = 0.0045 mol/nA.cm. Increasing values of α_*L*_ from top to bottom (see labels). The firing bistability displayed in A (top, α_*L*_ set to 0) persists when α_*L*_ is increased to 2% of α_*N*_, but the firing frequency is strongly reduced. When α_*L*_ is doubled, to 4% of α_*N*_, the abrupt transition to the low frequency state on the down ramp is replaced by a series of frequency plateaus reflecting mixed mode oscillations. **(C)** *F-I* curve for *G*_*Ca* − *L*_ = 0.5 mS/cm^2^. α_*L*_ set to 0 and α_*N*_ = 0.0045 mol/nA.cm as in A. Increasing values of *G*_AHP_ (see labels). Quiescence/firing bistability occurs for *G*_AHP_ smaller than 10 mS/cm^2^ (top), and the *F-I* curve is graded for larger *G*_AHP_. No firing bistability takes place. **(D)** Membrane currents. *G*_*Ca* − *L*_ = 1 mS/cm^2^ and *G*_AHP_ = 20 mS/cm^2^ (regime of firing bistability, see **A**). Top: L-type current (positive); bottom: sum of the leak and AHP currents (negative); dashed line: sum of the three currents. All currents are time averaged over the interspike interval and plotted as a function of the current injected in the soma. The zero current line is displayed in gray. The ascending current ramp is indicated by rightward pointing arrows and the descending branch by leftward pointing arrows.

Firing bistability can be achieved in this somatic model (see Figure 4A) but this requires that three conditions be satisfied. Firstly, the L-type current must activate during the voltage ramp preceding spikes and deactivate substantially during the AHP. Accordingly, we decreased the half-activation voltage of the L-type current from −40 to −55 mV to make it lower than the spike voltage threshold (−47 mV at the onset of the discharge), and we steepened its steady-state activation curve by decreasing the mid-activation voltage from 7 to 2 mV. For *G*_AHP_ = 20 mS/cm^2^, the activation of the L-type current then reached 75% after the first spike for *G*_*Ca* − *L*_ = 0.4 mS/cm^2^ and decayed to 7% at the end of the first interspike interval. This deactivation of the L-type current is crucial to ensure a strong dynamical competition with the AHP current.

Secondly, and importantly, the AHP conductance must be very little activated by calcium ions influx through L-type calcium channels, i.e., α_*L*_ must be much smaller than α_*N*_ in the evolution equation of the calcium concentration (see Materials and Methods). Otherwise, the AHP conductance is tonically activated and the discharge pattern dramatically altered, as illustrated in Figure 4B.

Thirdly, the L-type current must be sufficiently large, here again, as illustrated in Figure 4C for *G*_*Ca* − *L*_ = 0.5 mS/cm^2^. In the example shown, no firing bistability is achieved whereas firing bistability was observed for *G*_*Ca* − *L*_ = 1.0 mS/cm^2^ (see Figure 4A). Firing bistability actually requires that *G*_*Ca* − *L*_ be larger than 0.7 mS/cm^2^. For smaller *G*_*Ca* − *L*_, a direct transition from graded response to quiescence/firing bistability occurs.

Our results demonstrate that firing bistability may still occur when the L-type current is located at the soma. It then results from the dynamical competition between the L-type and AHP currents. This shows that segregation of the L-type current in the dendrites is not required for firing bistability. However, the dendritic location of the L-type current in motoneurons enhances firing bistability. In the somatic variant of our model, a large, and likely unrealistic, type-L conductance is needed to achieve firing bistability, and the hysteresis loop is distinctively smaller than in the dendritic variant studied in the preceding sections (compare Figures 3, 4).

The L-type and AHP currents respectively produce positive and negative feedbacks on the discharge. Figure 4D shows how the balance between these currents changes with the injected current in our somatic model. On the ascending branch of the ramp, the depolarizing L-type current (top) and the hyperpolarizing current (bottom), obtained by summing the AHP and leak currents, both increase with the injected current. Below 40 μA/cm^2^, the AHP current displays little saturation as the firing frequency increases and may successfully contain the activation of the L-type current. The L-type and hyperpolarizing currents remain approximately balanced, their sum never exceeding 3 μA/cm^2^ (dashed), and the firing rate increases at a low rate with the injected current. This is no longer possible when the injected current exceeds 40 μA/cm^2^. Then, the L-type current augments more than the hyperpolarizing current, their sum abruptly increases by 13 μA/cm^2^, and the model is pushed to the higher frequency state. On the descending branch of the ramp, the opposite scenario takes place. As long as the L-type current remains strongly activated, it maintains the discharge at an elevated frequency despite the negative feedback due to the AHP current. This happens until the injected current is decreased below 34 μA/cm^2^. The sum of currents then drops from 13 to −2 μA/cm^2^, and the model is pushed back to the low frequency state.

We performed a complete bifurcation analysis of this model but did not include it here to avoid technicalities. The mathematically oriented reader is invited to contact the corresponding author for full details (including bifurcation diagrams) about the bifurcation structure that underlies quiescence/firing bistability and firing bistability.

Altogether, our results suggest that firing bistability may occur whether the L-type current is located in the soma or in the dendrites and that it stems from the dynamical competition between L-type and AHP currents. Firing bistability occurs when both currents are large and is enhanced when the L-type current is located in dendrites.

### Experimental validation

We verified experimentally that bistability may indeed arise from the dynamical interaction between a somatic L-type current and the AHP current. Using dynamic clamp, an artificial L-type current was imposed through the recording microelectrode, which was, most likely, located at the soma. At variance with genuine L-type calcium currents, this artificial current provoked no calcium influx as the microelectrode was filled with KCl (see Methods). Therefore, it did not turn on the small conductance calcium-activated potassium channels (SK channels) responsible for the medium duration AHP. The AHP conductance was triggered only by calcium influx through high threshold calcium conductances during spikes, just like in our model. Thus, we turned limitations of the dynamic clamp methods (restriction to the soma, no calcium influx) to advantages.

**Figure 5:**
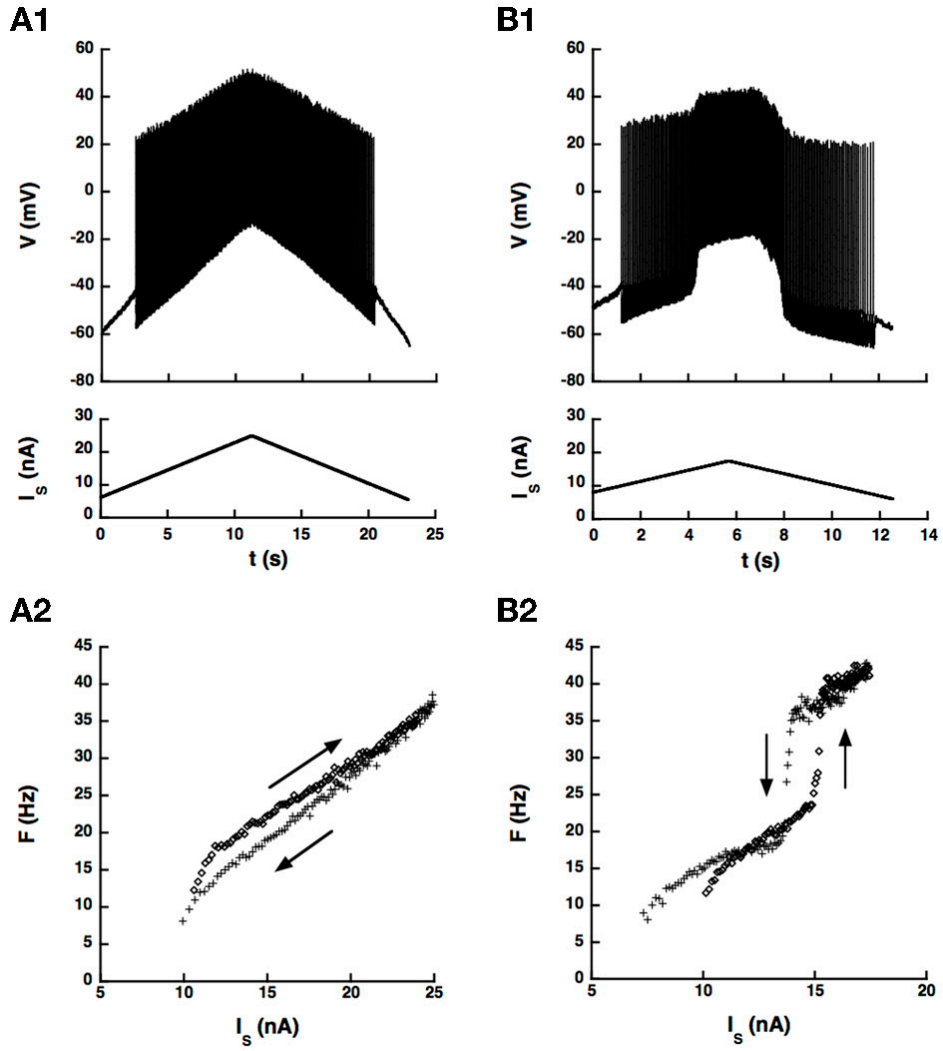
**An artificial L-type current injected in the soma can elicit a counterclockwise hysteresis in a spinal motoneuron**. CP motoneuron (axonal conduction velocity: 80 m/s, input conductance: 0.5 μ S). **(A)** No dynamic clamp current (control case). **(A1)** Ramp of current applied to the soma (bottom) and intracellular recording of soma voltage (top). **(A2)** F-I curve. The ascending branch (increasing current) is indicated by diamonds (upward arrow) and the descending branch (decreasing current) by crosses (downward arrow). **(B)** Same as A but a dynamic clamp current mimicking the L-type current was injected in the soma. Parameters of this current were the following. Conductance: 100 nS, half activation voltage: −35 mV, steepness of the activation curve: 2 mV, activation time constant: 50 ms, reversal potential: 80 mV.

We first checked whether the artificial L-type current could induce a hysteresis of the *F-I* curve in a sample of 14 motoneurons, mostly CP motoneurons. No hysteresis occurred in control condition in these cells as illustrated in Figure 5A, probably because the barbiturate used for anesthesia cats strongly depresses the natural calcium L-type current (Guertin and Hounsgaard, 1999). We recall that bistability has never been observed in anesthetized cats (Schwindt and Crill, 1982) but was shown to occur in decerebrate preparations where motoneurons were submitted to an intense monoaminergic neuromodulation (Hounsgaard et al., 1988; Lee and Heckman, 1998a,b, 1999). In contrast, when we added the artificial L-type current to the motoneuron of Figure 5A, we observed a clear counterclockwise hysteresis in the F-I relationship as shown in Figure 5B.

Altogether, the dynamic clamp current elicited a clear counterclockwise hysteresis in the *F-I* relationship of 8 motoneurons of our sample (7 CP, 1 Tib, 1 AbSm) but not in 6 others (5 CP and 1 Tib), at least in the range of parameters of the artificial current that we explored. The presence or absence of hysteresis is not a matter of motor pool. Indeed, most neurons recorded (86%) were CP motoneurons, 58% of which exhibited a hysteresis and 42% did not. In addition, hysteretic behavior was achieved only if the L-type current was large enough (conductance between 100 and 500 nS) and activated in the subthreshold voltage range traversed during the AHP after the first spike. This required that the half activation voltage of the L-type current be a few millivolts below the threshold for action potential generation (−4.5 ± 3 mV [−1 − −10 mV]), the most stringent condition to meet, and also that the steady-state activation curve was steep enough (*k_DC_* between 0.5 and 4.0 mV). Under these conditions, similar to those of our somatic model, the artificial L-type current was strongly modulated during the AHP, and a hysteresis was obtained over a wide range of activation time constant of the artificial L-type current, from 5 to 500 ms.

The observation of a hysteresis of the *F-I* curve in our experimental conditions is not sufficient to conclude that the recorded motoneurons exhibit genuine bistability. Indeed, the finite velocity (here, 2–5 nA /s depending on the motoneuron) of the current ramps enlarges the hysteresis loops and transforms the abrupt transitions from one firing state to the other into steep secondary ranges. Moreover, we cannot distinguish between long lasting up states (partial bistability over several seconds, see Lee and Heckman, 1998a,b) and genuinely stable up states (full bistability).

**Figure 6:**
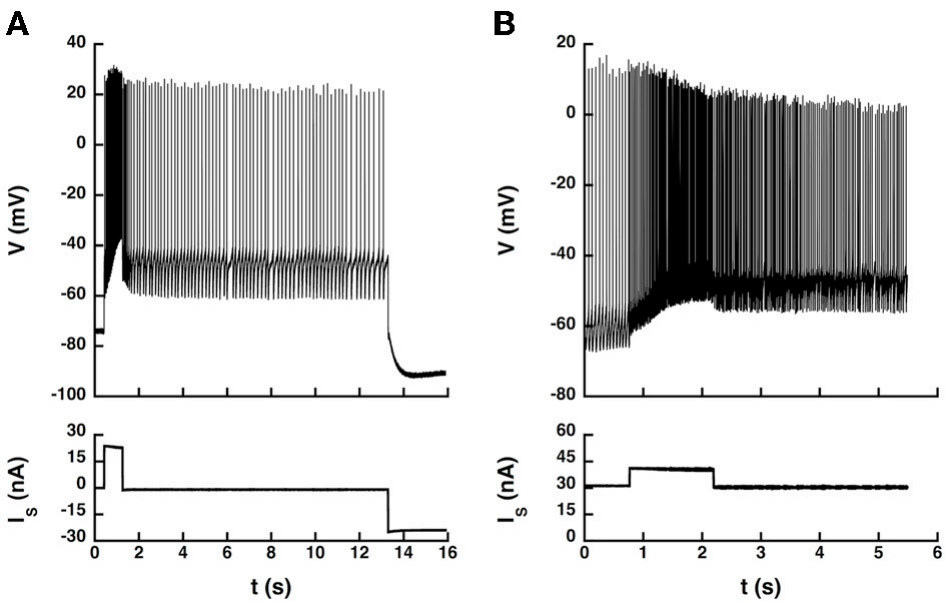
**Transitions between states elicited by current pulses. (A)** Quiescence/firing bistability. CP motoneuron (axonal conduction velocity: 90 m/s, input conductance: 1.0 μS). The current pulse (24 nA, 1 s) elicited an accelerating discharge that started with a frequency of 14 Hz and reached 50 Hz at the end of the pulse. After the pulse, the motoneuron kept firing at a lower frequency (5 Hz) instead of going back to rest. A hyperpolarizing current pulse (−24 nA) terminated firing. Artificial L-type current conductance: 350 nS, half-activation voltage: −50 mV, steepness of the activation curve: 2 mV, activation time constant: 400 ms. **(B)** Firing bistability. CP motoneuron (axonal conduction velocity: 90 m/s, input conductance: 1.1 μS). A 31 nA bias current was applied, which made the neuron steadily discharge at the mean frequency of 19 Hz. A pulse of 10 nA lasting 1.5 s was then superimposed to shift the motoneuron toward its up state (57 Hz, three times more than before the pulse). After the pulse, the motoneuron kept firing at a mean frequency of 30 Hz, about half more than the before the pulse. Two different firing states were thus obtained for the same injected current. The discharge in the up state (during and after the pulse) was more irregular than in the down state (before the pulse). L-type conductance: 820 nS, half-activation: −58 mV, steepness: 1 mV, activation time constant: 400 ms, reversal potential: 20 mV.

Therefore, we investigated whether current pulses elicited transitions between states in 5 of the 14 recorded motoneurons (4 CP and 1 ABSm). We used an artificial L-type current slower than the AHP by one order of magnitude, which induced bistability more easily. We provoked transitions between quiescence and firing in the ABSm motoneuron and in 2 CP motoneurons tested, as illustrated in Figure 6A, and we successfully triggered transitions between two different firing states in one of those two CP motoneurons, as illustrated in Figure 6B.

## Discussion

### Summary of results

We studied a model of motoneuron where the somatic and the dendritic compartments are 10 times more strongly coupled than in the BRK model. This is more in keeping with the location of the bulk of the L-type current in the dendrites of motoneurons. We demonstrated that the dynamical competition between the dendritic calcium L-type current and the somatic AHP current may lead to graded firing, quiescence/firing bistability below recruitment, or firing bistability, depending on the balance between these two currents. Firing bistability is achieved only for large intensities of the L-type current and when the AHP current also is sufficiently large to oppose the L-type current. In contrast, dendritic potassium currents play no other role than to decrease or suppress the negative slope region of the dendritic I–V curve.

Similar results were obtained when the L-type current was localized at the soma. In particular, firing bistability could still be achieved for large L-type current. However, this required that the AHP conductance be decoupled from the calcium L-type current, i.e., that it be triggered only by calcium ions influx through High Voltage Activated (HVA) conductances. This condition was naturally fulfilled in the dynamic clamp experiments in anesthetized cats that we performed to verify our theoretical predictions. Altogether, our results show that the dynamical interaction between the L-type current and AHP can create the couterclockwise hysteresis of the *F-I* curve. However, the spatial segregation that results from the different locations of the two currents in motoneurons (the L-type current mostly in dendrites and the AHP current in the soma) enhances that hysteresis.

### Somatic and dendritic components of the L-type currents

Most of the L-type current is found in the dendrites of motoneurons. Initially, the L-type current was thought to be located in distal dendrites, which explained the counterclockwise hysteresis of the I–V curve of motoneurons in response to a triangular voltage ramp in voltage clamp experiments. However, more recent studies suggest that the bulk of the L-type current is closer to the soma (0.6 ± 0.2 λ and spreads over a region where most synapses impinge (Elbasiouny et al., 2005, 2006; Bui et al., 2006; Grande et al., 2007). Booth et al. (1997) focused on the distal dendritic component of the L-type current and showed that firing bistability could emerge from the weak coupling between the distal dendrites and the axo-somatic region where spikes are initiated. In contrast, we focused in our two compartment model on the more proximal component of the L-type current, and we showed that firing bistability may result from the dynamical competition between the L-type current and the AHP current. In real motoneurons, there is of course no clear-cut separation between proximal and distal dendrites. Both scenarios likely contribute to bistability: the more proximal the L-type conductance is, the more it is coupled to the soma and the more its activation is controlled by the AHP.

Ballou et al. (2006) also showed that the calcium L-type current, although mostly dendritic, also displays a somatic component. We demonstrate that a somatic L-type current may also elicit quiescence/firing bistability and firing bistability in our model, provided it is large enough. This is not directly relevant for motoneurons, in which most of the L-type current is dendritic, but it indicates that the dynamical interaction of the L-type and AHP-currents *per se* is sufficient to produce firing bistability. This theoretical result was validated by dynamic clamp experiments.

Importantly, the control of the firing pattern by the interaction of the L-type and AHP currents requires in our model that somatic SK channels be mostly opened following the activation of the N-type calcium conductance. In contrast, dendritic potassium channels are essentially activated by calcium influx through L-type channels, HVA calcium channels being barely sensitive to the attenuated spikes that propagate back to the dendritic compartment. Our model thus suggests that motoneurons present two complements of SK channels, in the soma and in the dendrites, respectively activated by HVA and L-type calcium currents. Li and Bennett (2007) provided experimental evidence for that distinction. These authors indeed showed that the medium AHP was suppressed by the HVA calcium channel blocker ω-conotoxin but not by the L-type channel blocker nimodipine. In contrast, the dendritic calcium-sensitive potassium current that opposed the L-type current was eliminated by nimodipine.

### Conditions for firing bistability

Bistability between two firing states has been more rarely observed in motoneurons than between quiescence and firing. We suggest an explanation for that. In our model, firing bistability occurs when the following conditions are satisfied (i) the L-type current is large enough to elicit high frequency firing in the absence of AHP, (ii) the AHP current is also large and would create a wide primary firing range in the absence of L-type current, (iii) these two opposing currents are approximately balanced. Monaminergic neuromodulation of motoneurons increases the intensity of the L-type current (Hultborn and Kiehn, 1992). However, it also increases the overall excitability of motoneurons by reducing the AHP current. This is unfavorable to the balanced competition between the two currents required for firing bistability.

Firing bistability has been experimentally observed in motoneurons stimulated by a current injected in the soma. Elbasiouny et al. (2006) argued that firing bistability occurred only in that condition, whereas synaptic excitation of the dendrites led to secondary range firing at discharge onset. In our model, both current injection and synaptic excitation may elicit firing bistability when *G*_*Ca* − *L*_ is large enough, but the appropriate *G*_AHP_ range is narrower than for somatic current injection. Altogether, we may conclude that synaptic input to dendrites is indeed less favorable to firing bistability than somatic current injection.

### Differences with the BRK model

In the BRK model, spikes are attenuated by 96% in the dendrites because of the filtering by the small coupling conductance (0.1 mS/cm^2^) and the AHP is too small in dendrites (0.5 mV typically) to have a substantial impact on the activation of the L-type current. Increasing *G*_AHP_ extends the primary firing range (lower branch of the F-I curve), in keeping with the known role of the AHP (Kernell, 1968; Ermentrout, 1998; Manuel et al., 2006), but does not suppress firing bistability. As the current flowing from the soma is little modulated in time, the discharge properties can be deduced from the dendritic I–V curve. When the I–V curve is monotonic, the *F-I* curve is graded. In contrast, plateau potentials and bistability are observed when the I–V curve is N-shaped. When the I–V curve intersects only once the zero current axis, a high frequency discharge occurs right from firing onset, and quiescence/firing bistability is achieved below recruitment. When there are three intersections, firing bistability is observed in response to a triangular current ramp, alone or together with quiescence/firing bistability.

In our model, where the two compartments are more strongly coupled (1 mS/cm^2^), the AHP is barely reduced in the dendrites and may deactivate the L-type current. Increasing sufficiently the AHP leads to graded firing. This cannot be explained by a change in the I–V curve. Indeed, increasing *G*_AHP_ does alter the I–V curve in the suprathreshold voltage range but does not eliminate the negative slope region created by the L-type current below threshold. The interaction between the L-type and AHP currents is a dynamical effect that occurs during firing and controls the firing pattern. It could not be grasped by the BRK model, in which the L-type current was located distally in dendrites in a region unaffected by the AHP and bistability emerged from the plateau properties of the dendritic compartment.

At variance with the BRK model, our model does not incorporate a distal L-type current, which is why the somatic I–V curve displays no counterclockwise hysteresis. As a result, the L-type current is larger in our model than was estimated in motoneurons of decerebrate cats (see, for instance, Lee and Heckman, 1998a,b). This is particularly true of the somatic component. It is likely that the introduction of a distal component of the L-type current, in addition to the proximal component considered in our study, would allow bistable behavior for weaker L-type current.

### The interaction between the L-type and AHP currents is crucial for the control of motoneuron discharge

Motoneurons exhibit a large AHP that plays a fundamental role in controlling their firing pattern. It is well established that the AHP limits the frequency and the variability of their discharge (Kernell, 1999; Powers and Binder, 2000; Manuel et al., 2006). The AHP also affects excitability by interacting with inward currents. For instance, we recently showed that the AHP suppressed the mixed mode oscillations associated with subprimary range firing in mouse motoneurons (Iglesias et al., 2011) by deinactivating the sodium current, thus increasing membrane excitability. In the present study, the AHP decreases the excitability by deactivating the L-type current, which is in line with the modeling study of Elbasiouny et al. (2006). This did not occur in the BRK model because the coupling was too weak to allow the AHP to interact with the distal dendritic L-type current. Altogether, the firing properties of motoneurons appear to be largely regulated by the interaction between the AHP and L-type currents.

### Conflict of interest statement

The authors declare that the research was conducted in the absence of any commercial or financial relationships that could be construed as a potential conflict of interest.

## References

[B1] Ballou E. W. Smith W. B. Anelli R. Heckman C. J. (2006). Measuring dendritic distribution of membrane proteins. J. Neurosci. Methods. 156, 257–266 10.1016/j.jneumeth.2006.03.014 16690134

[B2] Booth V. Rinzel J. Kiehn O. (1997). Compartmental model of vertebrate motoneurons for Ca2+-dependent spiking and plateau potentials under pharmacological treatment. J. Neurophysiol. 78, 3371–3385 9405551 10.1152/jn.1997.78.6.3371

[B3] Brizzi L. Meunier C. Zytnicki D. Donnet M. Hansel D. Lamotte d'Incamps B. (2004). How shunting inhibition affects the discharge of lumbar motoneurones: a dynamic clamp study in anaesthetized cats. J. Physiol. 558(Pt 2), 671–683 10.1113/jphysiol.2003.059964 15169842 PMC1664972

[B4] Bui T. V. Ter-Mikaelian M. Bedrossian D. Rose P. K. (2006). Computational estimation of the distribution of L-type Ca(2+) channels in motoneurons based on variable threshold of activation of persistent inward currents. J. Neurophysiol. 95, 225–241 10.1152/jn.00646.2005 16267115

[B5] Elbasiouny S. M. Bennett D. J. Mushahwar V. K. (2005). Simulation of dendritic CaV1.3 channels in cat lumbar motoneurons: spatial distribution. J. Neurophysiol. 94, 3961–3974 10.1152/jn.00391.2005 16120667

[B6] Elbasiouny S. M. Bennett D. J. Mushahwar V. K. (2006). Simulation of Ca2+ persistent inward currents in spinal motoneurones: mode of activation and integration of synaptic inputs. J. Physiol. 570, 355–374 10.1113/jphysiol.2005.099119 16308349 PMC1464303

[B7] Ermentrout B. (1998). Linearization of F-I curves by adaptation. Neural Comput. 10, 1721–1729 10.1162/089976698300017106 9744894

[B8] Fleshman J. W. Segev I. Burke R. B. (1988). Electrotonic architecture of type-identified alpha-motoneurons in the cat spinal cord. J. Neurophysiol. 60, 60–85 3404225 10.1152/jn.1988.60.1.60

[B9] Grande G. Bui T. V. Rose P. K. (2007). Estimates of the location of L-type Ca2+ channels in motoneurons of different sizes: a computational study. J. Neurophysiol. 97, 4023–4035 10.1152/jn.00044.2007 17428909 PMC2930907

[B10] Guertin P. A. Hounsgaard J. (1999). Non-volatile general anaesthetics reduce spinal activity by suppressing plateau potentials. Neuroscience 88, 353–358 10.1016/S0306-4522(98)00371-6 10197758

[B11] Hounsgaard J. Hultborn H. Jespersen B. Kiehn O. (1988). Bistability of α-motoneurones in the decerebrate cat and in the acute spinal cat after intravenous 5-hydroxytryptophan. J. Physiol. 405, 345–367 3267153 10.1113/jphysiol.1988.sp017336 PMC1190979

[B12] Hounsgaard J. Kiehn O. (1985). Ca^++^ dependent bistability induced by serotonin in spinal motoneurons. Exp. Bain Res. 57, 422–425 10.1007/BF00236551 2578974

[B13] Hounsgaard J. Kiehn O. (1989). Serotonin-induced bistability of turtle motoneurones caused by a nifedipine-sensitive calcium plateau potential. J. Physiol. 414, 265–282 2607432 10.1113/jphysiol.1989.sp017687 PMC1189141

[B14] Hounsgaard J. Mintz I. (1988). Calcium conductance and firing properties of spinal motoneurones in the turtle. J. Physiol. 398, 591–603 2455804 10.1113/jphysiol.1988.sp017059 PMC1191789

[B15] Hultborn H. Kiehn O. (1992). Neuromodulation of vertebrate motor neuron membrane properties. Curr. Opin. Neurobiol. 2, 770–775 10.1016/0959-4388(92)90132-5 1282406

[B16] Iglesias C. Meunier C. Manuel M. Timofeeva Y. Delestrée N. Zytnicki D. (2011). Mixed mode oscillations in mouse spinal motoneurons arise from a low excitability state. J. Neurosci. 31, 5829–5840 10.1523/JNEUROSCI.6363-10.2011 21490224 PMC6622841

[B17] Jaffe D. Carnevale N. (1999). Passive normalization of synaptic integration influenced by dendritic architecture. J. Neurophysiol. 82, 3268–3285 10601459 10.1152/jn.1999.82.6.3268

[B18] Kernell D. (1968) The repetitive impulse discharge of a simple neurone model compared to that of spinal motoneurones. Brain Res. 11, 685–687 10.1016/0006-8993(68)90157-1 5712015

[B19] Kernell D. (1999). Repetitive impulse firing in motoneurons: facts and perspectives. Prog. Brain Res. 123, 31–37 10.1016/S0079-6123(08)62841-1 10635701

[B20] Kim H. Major L. Jones K. (2009). Derivation of cable parameters for a reduced model that retains asymmetric voltage attenuation of reconstructed spinal motor neuron dendrites. J. Comput. Neurosci. 27, 321–336 10.1007/s10827-009-0145-7 19387812

[B21] Kuo J. J. Lee R. H. Zhang L. Heckman C. J. (2006). Essential role of the persistent sodium current in spike initiation during slowly rising inputs in mouse spinal neurones. J. Physiol. 574(Pt 3), 819–834 10.1113/jphysiol.2006.107094 16728453 PMC1817738

[B22] Lee R. H. Heckman C. J. (1998a). Bistability in spinal motoneurons *in vivo*: systematic variations in rhythmic firing patterns. J. Neurophysiol. 80, 572–582 9705451 10.1152/jn.1998.80.2.572

[B23] Lee R. H. Heckman C. J. (1998b). Bistability in spinal motoneurons *in vivo*: systematic variations in persistent inward currents. J. Neurophysiol. 81, 2164–2174 9705452 10.1152/jn.1998.80.2.583

[B24] Lee R. H. Heckman C. J. (1999). Enhancement of bistability in spinal motoneurons *in vivo* by the noradrenergic alpha1 agonist methoxamine. J. Neurophysiol. 80, 583–593 10322057 10.1152/jn.1999.81.5.2164

[B25] Li X. Bennett D. J. (2007). Apamin-sensitive calcium-activated potassium currents (SK) are activated by persistent calcium currents in rat motoneurons. J. Neurophysiol. 97, 3314–3330 10.1152/jn.01068.2006 17360829 PMC5718199

[B26] Li Y. Gorassini M. A. Bennett D. J. (2004). Role of persistent sodium and calcium currents in rat motoneuron firing and spasticity in chronic spinal rats. J. Neurophysiol. 91, 767–783 10.1152/jn.00788.2003 14762149

[B27] Manuel M. Iglesias C. Donnet M. Leroy F. Heckman C. J. Zytnicki D. (2009). Fast kinetics, high-frequency oscillations, and subprimary firing range in adult mouse spinal motoneurons. J. Neurosci. 29, 11246–11256 10.1523/JNEUROSCI.3260-09.2009 19741131 PMC2785440

[B28] Manuel M. Meunier C. Donnet M. Zytnicki D. (2006). The afterhyperpolarization conductance exerts the same control over the gain and variability of motoneurone firing in anaesthetized cats. J. Physiol. 576(Pt 3), 873–886 10.1113/jphysiol.2006.117002 16931549 PMC1890407

[B29] Moritz A. T. Newkirk G. Powers R. K. Binder M. D. (2007). Facilitation of somatic calcium channels can evoke prolonged tail currents in rat hypoglossal motoneurons. J. Neurophysiol. 98, 1042–1047 10.1152/jn.01294.2006 17522175

[B30] Powers R. K. Binder M. D. (2000). Relationship between the time course of the afterhyperpolarization and discharge variability in cat spinal motoneurones. J. Physiol. 528, 131–150 10.1111/j.1469-7793.2000.t01-1-00131.x 11018112 PMC2270116

[B31] Prinz A. A. Abbott L. F. Marder E. (2004). The dynamic clamp comes of age. Trends Neurosci. 27, 218–224 10.1016/j.tins.2004.02.004 15046881

[B32] Raikov I. Preyer A. Butera R. J. (2004). MRCI: a flexible real-time dynamic clamp system for electrophysiology experiments. J. Neurosci. Methods 132, 109–123 10.1016/j.jneumeth.2003.08.002 14706709

[B33] Schwindt P. C. Crill W. E. (1982). Factors influencing rhythmic firing: results fromm a voltage-clamp study. J. Neurophysiol. 48, 875–890 7143033 10.1152/jn.1982.48.4.875

[B34] Schwindt P. C. Crill W. E. (1984). Membrane properties of cat spinal motoneurons in Handbook of the Spinal Cord, ed Davidoff R. (New York, NY: Dekker), 199–242

[B35] Simon M. Perrier J. F. Hounsgaard J. (2003). Subcellular distribution of L-type Ca2+ channels responsible for plateau potentials in motoneurons from the lumbar spinal cord of the turtle. Eur. J. Neurosci. 18, 258–266 10.1046/j.1460-9568.2003.02783.x 12887407

[B36] Zhang M. Møller M. Broman J. Sukiasyan N. Wienecke J. Hultborn H. (2008). Expression of calcium channel CaV1.3 in cat spinal cord: light and electron microscopic immunohistochemical study. J. Comp. Neurol. 507, 1109–1127 10.1002/cne.21595 18095323

[B37] Zhang M. Sukiasyan N. Møller M. Bezprozvanny I. Zhang H. Wienecke J. (2006). Localization of L-type calcium channel Ca(V)1.3 in cat lumbar spinal cord with emphasis on motoneurons. Neurosci. Lett. 407, 42–47 10.1016/j.neulet.2006.07.073 16949207

